# Tailoring and optimization of a honey-based nanoemulgel loaded with an itraconazole–thyme oil nanoemulsion for oral candidiasis

**DOI:** 10.1080/10717544.2023.2173337

**Published:** 2023-01-27

**Authors:** Amal M. Sindi, Waleed Y. Rizg, Muhammad Khalid Khan, Hala M. Alkhalidi, Waleed S. Alharbi, Fahad Y. Sabei, Eman Alfayez, Hanaa Alkharobi, Mohammed Korayem, Mohammed Majrashi, Majed Alharbi, Mohammed Alissa, Awaji Y. Safhi, Abdulmajeed M. Jali, Khaled M. Hosny

**Affiliations:** a Department of Oral Diagnostic Sciences, Faculty of Dentistry, King Abdulaziz University, Jeddah, Saudi Arabia; b Department of Pharmaceutics, Faculty of Pharmacy, King Abdulaziz University, Jeddah21589, Saudi Arabia; c Department of Biochemical Materials, Beautsway commercial foundation, Cairo, Egypt; d Department of Clinical Pharmacy, Faculty of Pharmacy, King Abdulaziz University, Jeddah, 21589, Saudi Arabia; e Department of Pharmaceutics, College of Pharmacy, Jazan University, Jazan45142, Saudi Arabia; f Department of Oral Biology, Faculty of Dentistry, King Abdulaziz University, Jeddah, Saudi Arabia; g Preventive Dental Sciences Department, Faculty of Dentistry, Albaha University, Albaha, Saudi Arabia; h Department of Pharmacology, College of Medicine, University of Jeddah, Jeddah, 23890, Saudi Arabia; i Department of Pharmaceutical Chemistry, Faculty of Pharmacy, King Abdulaziz University, Jeddah, Saudi Arabia; j Department of Medical Laboratory Sciences, College of Applied Medical Sciences, Prince Sattam bin Abdulaziz University, Al-Kharj, 11942, Saudi Arabia; k Department of Pharmacology and Toxicology, College of Pharmacy, Jazan University, Saudi Arabia

**Keywords:** Sustainability of natural resources, Honey-based gel, Itraconazole, thyme oil, design of experiments, oral microbiota

## Abstract

The use of essential oil–based nanoemulsions (NEs) has been the subject of extensive research on a variety of conditions affecting the oral cavity. NEs are delivery methods that improve the solubility and distribution of lipid medicines to the intended areas. Because of their antibacterial and antifungal properties, itraconazole and thyme oil–based self-nanoemulsifying drug delivery systems (ItZ-ThO-SNEDDS) were created to protect oral health against oral microorganisms. The ItZ-ThO-SNEDDS were created utilizing an extreme verices mixture design, and varying concentrations of ThO (10% and 25%), labrasol (40% and 70%), and transcutol (20% and 40%) were used. The ItZ-ThO-SNEDDS had droplet sizes of less than 250 nm, a drug-loading efficiency of up to 64%, and a fungal growth inhibition zone of up to 20 mm. The accepted design was used to obtain the ideal formulation, which contained ThO in the amount of 0.18 g/ml, labrasol 0.62 g/ml, and transcutol 0.2 g/ml. The best ItZ-ThO-SNEDDS formulation was incorporated into a honey-based gel, which demonstrated improved release of ItZ *in vitro* and improved transbuccal permeation *ex vivo*. In addition, when compared with various formulations tested in rats, the optimized loaded emulgel decreased the ulcer index. This study therefore demonstrated that the ItZ-ThO-SNEDDS could offer an effective defense against oral diseases caused by microbial infections.

## 1. Introduction

For overall health and a high quality of life, oral health is crucial. In 2012, the World Health Organization defined *oral health* as the absence of mouth or facial pain, oral infections or sores, and other disorders that restrict a person’s ability to engage in with his daily and psychosocial activities (World Health Organization, 2012). Oral diseases continue to be a neglected area of global health despite the significant social and financial costs they impose on many nations (FDI World Dental Federation, 2015). Most adults have tooth decay, and 15% to 20% of middle-aged adults have serious gum disease (Buset et al., 2016). Poor dental health may be linked to such diseases as diabetes, heart disease, stroke, pneumonia, and other respiratory illnesses, according to the literature (Silva et al., 2015). Another typical reason for headaches and ear and facial pain is disorders of the jaw (Katz et al., 2010). Dental clearance prior to medical procedures such as cardiac surgery, cancer/bisphosphonate therapy, and radiation therapy reduces the systemic and oral side effects of these procedures (Rajan et al., 2014).

Free-living eukaryotic organisms known as fungi can be molds (filamentous fungi), yeasts (round fungi), or a blend of both (dimorphic fungi) (Terezhalmy & Huber, 2011). One of the common fungal infections of the oral mucus membranes is oral candidiasis (Prasanna, 2012). It is caused by the yeast *Candida albicans,* which is a fairly normal component of the oral microbiota; it is found in 30% to 50% of people. As people age, the rate of carriage rises (Dangi et al., 2010). In 60% of patients over 60 years of age with dental problems, *C. albicans* is recovered from their mouths (Parihar, 2011). In addition to *C. albicans*, there are several other species of *Candida* that can be found in the oral environment, including *Candida guilliermondii, Candida krusei, Candida glabrata, Candida parapsilosis, Candida pseudotropicalis, and Candida tropicalis* (Dangi et al., 2010).

Itraconazole (ItZ) is a triazole antifungal that can be taken orally and has a propitious pharmacokinetic profile. It has been shown to have a wide range of efficacy (Chong & Sullivan, 2007). ItZ has been demonstrated in noncomparative clinical trials to be highly effective when delivered once or twice daily for a variety of superficial and more consequential ‘deep’ fungal ailments (DiMasi et al., 2003). This drug’s original purpose was to act as a broad-spectrum antifungal drug by inhibiting 14-α-lanosterol demethylase (14LDM), an enzyme that causes ergosterol to be produced in fungi and cholesterol to be produced in humans (Okabayashi et al., 2009). It is used to prevent immunosuppressive diseases, as well as treat fungal infections such aspergillosis, candidiasis, and histoplasmosis (Chang et al., 2017). ItZ has uncommon side effects such as neutropenia, liver failure, and heart failure but is generally considered to be safe (Girois et al., 2006).

There are numerous members of the genus *Thymus* of the family Lamiaceae. These plants are frequently employed for culinary, cosmetic, and therapeutic purposes (Ghasemi et al., 2020). The most recognized plant ingredient in the industrial pharmacy sector is thyme, which is derived from *Thymus vulgaris* L. and *Thymus zygis* L. (Kosakowska et al., 2020). The chemical constituents of thyme essential oil (EO) include monoterpene alcohols, phenol derivatives, ketones, aldehydes, ethers, and esters, among other chemical groups (Bendif et al., 2020). The two isomeric phenolic monoterpenes thymol (2-isopropyl-5-methylphenol) and carvacrol (2-methyl-5-(1-methyethyl) phenol) make up most of the thyme EOs (Sharifi-Rad et al., 2018; Nagoor Meeran et al., 2017).

For centuries, people have used the thyme plant and its EOs to treat infections related to the upper respiratory tract, the symptoms of bronchitis, pruritus brought on by dermatitis, bruises, and sprains (Kohlert et al., 2002). These days, it is frequently used in dental medicine as a disinfectant and as an expectorant for coughs brought on by colds (Nilima et al., 2013). It has antibacterial effects on both Gram-positive and Gram-negative bacteria, as well as antifungal, antispasm, anti-inflammatory, antioxidant, and antiviral activities against human rhinoviruses, influenza viruses, and herpes simplex virus type I (Walther et al., 2020). There have been no cases of toxicity when the oil is taken at levels regularly used, and therefore it is generally regarded as safe (Eva et al., 2018; Salehi et al., 2018).

Several antimicrobial medicines have lost their efficacy for treating wounds owing to the development of treatment-resistant microbes (Guo & Dipietro, 2010). The use of honey for wounds is one of the alternative medicines that have been sought out more widely used in clinical settings for the treatment of wounds (Molan, 2001). Honey’s inherent qualities and active ingredients are essential for the process of healing in wounds (Molan & Rhodes, 2015). Natural honey has a jelly-like viscosity and forms a superficial film covering wounds and lesions and prevents bacteria and dehydration (Langemo et al., 2009; Sell et al., 2012). Because of its high sugar content, honey produces a strong osmotic gradient that draws fluid up the subdermal tissue and provides a source of glucose for the thriving cellular components in wounds (Molan, 2002). Honey’s water activity is lower than 0.91 aw, and this inhibits and limits bacterial development on injured sites and promotes fluid movement that causes debris and necrotic tissue to be sloughed from the wound (Gleiter et al., 2006; Sundoro et al., 2012).

A breakthrough idea called nanomedicine has shown great promise for delivering prospective medications with low solubility and bioavailability (Salem et al., 2022; Hussein et al., 2022; Ali et al., 2021; Alhakamy et al., 2022). A nanoemulsion (NE) is a delivery system that can improve solubility and stability (Rizg et al., 2021). Widely researched as drug delivery platforms, NEs are made up of nanosized oil droplets stabilized by a combination of surfactants and co-surfactants (Hosny et al., 2021). Using EO–based NEs to combat oral bacteria is a viable strategy (Hosny et al., 2021). Gels are relatively new dosage forms often generated when significant quantities of aqueous, hydroalcoholic, or nonpolar vehicles are mixed in a three-dimensional (3D) network of polymeric components (Ali et al., 2021). However, they are less effective at delivering hydrophobic medications (Wong & Dodou, 2017). Emulgels, a hybrid of emulsions and gels, were therefore a viable alternative that would allow introducing hydrophobic medications topically (Talat et al., 2021). These dosage forms are well liked because they combine the benefits of both emulsions and gels, like regulated drug release of emulsions and excellent thermodynamic stability of gels (Laffleur & Keckeis, 2020).

In order to gather the most data with the fewest experiments, and provide an explanation for any experimental errors, statistical experimental designs were developed (Gregg Stetsko Manager, 1986). Utilizing these designs has the added benefit of requiring that formulations be made carefully and that statistical laws be followed. This means that scientists must be precise when setting up the experimental goals and the procedures for meeting those goals. Additionally, the design can forecast the behavior of the ideal formulation (Hosny et al., 2020). Because statistical designs frequently lead to an optimum formulation, they can be a cost-effective method of study (Alkhalidi et al., 2018). In order to create a novel formulation for the successful treatment of oro-mucosal ulcers brought on by oral candidiasis, the current study targeted the development of a honey-based nanoemulgel containing an itraconazole and thyme oil–based nanoemulsion (ItZ-ThO-NE) and evaluated its antibacterial and antifungal efficacy and calculated its ulcer index.

## 2. Materials and methods

### 2.1. Materials

The beauts-way Commercial Co., Ltd. (Jeddah, Saudi Arabia) provided the ItZ and ThO. The Saudi Arabian Japanese Pharmaceutical Company Ltd generously donated the PVP and PEG (SAJA, Jeddah, Saudi Arabia). Agar and Laureth-20 were acquired from the Sigma Water Import and Export Co., Ltd. (Shaanxi, China). Tween and Cremophor EL, as well as isopropyl alcohol and glycerin, were procured from Sigma Aldrich (St. Louis, Missouri, USA). Labrasol and transcutol were generously provided by Gattefosse (Saint-Priest, France), and the honey was acquired from the Hali Health Company (Ras Al Khaimah, UAE). The other compounds that were utilized in this experiment were all of analytical grade. Purified water was used in the experiments.

### 2.2. Methods

#### 2.2.1. Solubility studies

To determine the variable self-emulsifying regions that may be used to construct ThO-based NEs containing ItZ, the drug solubility was evaluated in a variety of surfactants and co-surfactants (Hosny et al., 2021).

To determine the drug’s solubility in each specific solution, excess quantities of ItZ were dissolved in 3 ml of the surfactants, namely, Tween, labrasol, Laureth-20, and Cremophor EL, as well as the co-surfactants transcutol, isopropyl alcohol, glycerin, HCO-20, and propylene glycol. The combinations were kept in vials for 3 days at 25 ± 2 °C in a water bath. After the required balance was achieved, the mixtures were precipitated by centrifugation for 20 minutes at 4000 rpm to remove any extra medication. In order to determine the ItZ content of the supernatants, a high-performance liquid chromatography (HPLC) method using a C18 HS (250 × 4.6 mm) column was employed (Thimmaraju et al., 2012). Acetonitrile and double-distilled water were combined in this procedure’s mobile phase in a ratio of 90:10 v/v at a flow rate of 1.0 ml/min. A UV detector set at 263 nm was used for the detection. It was discovered that ItZ had a retention time of 7.5 minutes. ItZ also had a linearity range of 4 to 60 µg/ml and a linear regression coefficient of 0.991.

#### 2.2.2. Itz-ThO emulsifying regions’ tenacity in particular surfactants and co-surfactants

In a review to determine the lower and higher limits for every ingredient of the self-nanoemulsifying drug delivery systems (SNEDDS), which were chosen based on solubility, the self-emulsifying regions were located using pseudoternary phase diagrams. When diluted with water and gently stirred within those limits, blends of ItZ, ThO, the chosen surfactant (labrasol), and the chosen co-surfactant (transcutol) spontaneously created a pellucid emulsion with globules in the nanometer range. When the droplet size is greater than 1 nm but smaller than 1 m, the emulsion area in the phase area can be recognized as an NE region. The ItZ-loaded NEs were then tested.

#### 2.2.3. Design of ItZ-ThO self-nanoemulsifying formulations and optimization of experiments

Because of its great effectiveness in evaluating the emulsions and projecting the best solutions, an extreme vertices mixture experimental design (Table 1) was used for the creation and selection of the best ItZ-ThO-SNEDDS. To ascertain the impacts of the ThO level (A), labrasol level (B), and transcutol level (C) in 17 formulations, a mixed design based on extreme vertices was used. The prepared SNEDDS’ globule size (Y_1_), drug-loading efficiency (Y_2_), and growth inhibition of *C. albicans* (Y_3_) were the dependent responses. The response factors were noted and optimized utilizing the Design of Experiments.

**Table 1.** Upper and lower limits of investigated factors combined with measured responses and their constraints.

Studies factors	Levels	
	low	high
A: ThO %	10.0	25.0
B: Labrasol %	40.0	70.0
C: Transcutol %	20.0	40.0
Measured responses	Constrains	
Droplet size (Y_1_)	minimize	
ItZ loading capability (Y_2_)	Maximize	
Inhibition Zone (Y_3_)	Maximize	

#### 2.2.4. Visual screening of ItZ-ThO-SNEDDS

Visual inspection was used to assess the effectiveness of the production of the ItZ-ThO-SNEDDS in terms of their clarity and stability; this included defects such as coalescence or the shattering of spontaneously generated NEs (Hosny et al., 2021).

#### 2.2.5. Assessment of the nanoemulsions’ droplet sizes

Double-distilled deionized water, 400 ml, was used to dilute 100 ml of each formulation, and the blend was mixed using a vortex mixer for 5 minutes. Aliquots of 200 µl were taken from the diluted samples to determine the droplet size and polydispersity index (PDI) using a Zetatrac and a dynamic light-scattering approach (Microtrac, Inc., Montgomeryville, Pennsylvania, USA) (Hosny et al., 2021).

#### 2.2.6. Analysis of ItZ-ThO-SNEDDS’ drug-loading efficiency

By independently dispersing a known excess quantity of ItZ in 1 g of a plain SNEDDS, the ItZ loaded in each SNEDDS mixture was calculated. The mixtures were placed in vials and kept in a shaking water bath at 25 °C for 24 hours. The blends were precipitated by centrifugation for approximately 15 minutes at 4500 rpm after reaching equilibrium. The precipitates were gathered, thoroughly cleaned, and then dissolved in methanol. The amount of ItZ was calculated using the previously described HPLC procedure after the drug had been removed. The following mathematical formula was used to calculate the drug-loading capacity (Hosny et al., 2021):

Eqn. (1) $$\text{Drug loading efficiency }=\;\frac{\mathrm{Produc}t^{\prime }s\;\mathrm{drug}\;\mathrm{content}\;(mg)}{\mathrm{Total}\;\mathrm{product}\;\mathrm{weight}\;(mg)}\;\times 100$$

#### 2.2.7. Valuation of growth inhibition zone against Candida albicans

The disk diffusion method was used to assess the formulation’s antifungal effectiveness. To prepare *C. albicans* strains for inoculation and conduct an *in vitro* antifungal evaluation utilizing the fungus strains, the strains were grown on Sabouraud dextrose agar medium (Hosny et al., 2021).

##### 2.2.7.1. Inoculum development:

Five standard *C. albicans* strain colonies were chosen, suspended in 2 ml of normal saline, and thoroughly mixed. The 0.6-meq McFarland standards were used to preserve the uniform turbidity of the microbial suspension. To obtain the grass culture, streaks were produced on the Sabouraud dextrose agar media by inserting a sterilized cotton swab into the prepared fungal suspension.

##### 2.2.7.2. Disk diffusion technique:

To prepare the test and positive control samples, sterile filter paper disks measuring 5 mm in diameter were submerged in 100 µl of the ItZ-ThO-SNEDDS and ethyl alcohol (99.9%), respectively. The paper disks were inserted into seeded Sabouraud dextrose agar media after being soaked. The infected plates were incubated for 48 hours at 24 ± 2 °C before they were examined for each zone of inhibition.

#### 2.2.8. Optimization of ItZ-ThO-SNEDDS

For the analysis of variance (ANOVA) of the resulting models, the *F*-ratio, *p*-value, and degrees of freedom were computed for the investigated parameters and their interactions. The responses were utilized to choose the model that would be most suitable for the data based on the results. The *p*-values below .05 showed that the tested model predictors were significant. The model fitness was further evaluated using the coefficient of variation (CV%) values, determination correlations, anticipated R^2^ value, and adapted R^2^ value. In this way, the NEs with the smallest globule size and highest drug-loading efficacy were identified.

#### 2.2.9. Choosing the optimized ItZ-ThO-SNEDDS

The development and evaluation of the formulations involved evaluating the drug-loading capability and globule size. The formulations were then examined for their *ex-vivo* permeation, *in-vitro* drug release, and ulcer index.

#### 2.2.10. Preparation of honey-based nanoemulgel loaded with ItZ-ThO-NE

An optimized ItZ-ThO-SNEDDS–loaded honey nanoemulgel was created by combining 5% (v/v) honey with 10% (w/v) PVP, 1% (w/v) agar solution, and 2% (v/v) PEG. An aqueous PVP solution made by dispersing 10 g of PVP in 60 ml of distilled water was used to create the simple honey gel (i.e. formulation F1), which was allowed to sit at room temperature (25 °C) overnight. To create the agar blend, 1 g of agar was solubilized in 20 ml of distilled water, heated until clear, and stirred constantly before 2 ml of PEG was added. Fifteen ml of honey was introduced when the mixture’s temperature dropped below 45 °C after the homogeneous mixture had been in a sonicator water bath for an hour at 37 °C. The same procedures were followed in the preparation of an optimized honey nanoemulgel (i.e. formulation F2) that was loaded with the optimized ItZ-ThO-SNEDDS, but 10 g of PVP was dissolved in 60 ml of the NE created by dissolving 10 ml of the ItZ-ThO-SNEDDS in 50 ml of distilled water and stirring for 5 minutes before adding PVP. The remaining ingredients were then added as in the previously prepared plain honey gel.

Additionally, four different hydrogel formulations were prepared for comparison (formulations F3 to F6). ItZ as a powder and ThO as an oil were used to make the honey gel for F3; that is, a SNEDDS was not used. The nanoemulgel for F4 was created without adding honey. The F5 honey gel, manufactured in the same way as the F3, did not contain ThO, and the F6, prepared in the same way as the F4, did not contain ItZ powder. The produced gels were evaluated for the following characteristics.

##### 2.2.10.1. In-vitro dissolution of the hydrogel formulations

In this study, the USP Dissolution Tester (Apparatus I) was used. Instead of using baskets, glass cylindrical tubes measuring 2.7 cm in diameter and 10 cm in length were attached to the spinning shafts and securely sealed with semipermeable membranes of 100 µm in pore size. These tubes contained the formulations to be assessed. The tested formulations (F2 to F5) each weighed 10 g and contained 50 mg of ItZ. Each glass tube containing one of the formulations was submerged in 50 ml of phosphate buffered saline (PBS, pH 6.8). The medium was agitated at a speed of 25 rpm while the release study was conducted at 37 ± 0.5 °C.

Over 1 hour, samples were taken out of the dissolved mixture at various intervals. Using the previously described HPLC procedure, the absorbance was measured to determine how much ItZ had been released (Hosny et al., 2021).

##### 2.2.10.2. Ex-vivo transmucosal penetration test of the hydrogel formulations

*Ex-vivo* tests were conducted on the ItZ aqueous suspension and various manufactured formulations (F2 to F5), each of which contained 5 mg/ml of ItZ. An automated Franz diffusion cell (MicroettePlus, Scilogex LLC, Rocky Hill, Connecticut, USA) was used as the apparatus, and sheep buccal mucosal membrane obtained from a nearby local butcher was used as the model penetration membrane. The Franz diffusion cell’s donor and receptor compartments were adequately separated by the processed sheep buccal mucosa. The exposed surface area of the receptor compartment covered by the membrane was 1.75 cm^2^. The medium was agitated at a rate of 400 to 450 rpm while being kept at a temperature of 37 ± 0.5 °C in the drawing chamber, which encompassed 8 ml of PBS (pH 6.8).

At regular intervals, precise aliquots were automatically removed, and the previously described HPLC method was used to quantify the ItZ content. A graph was made to show how long it took for the ItZ to penetrate the Q per cm^2^ of the membrane; this finding let researchers better understand how the drug was dispersed throughout the mucosa. From the acquired diffusion data, significant parameters such as the Jss (steady-state flow), Pc (permeability coefficient), EF (enhancement factor), and D (diffusion coefficient) were determined. Plots were made of the comparative dissemination patterns for various formulations. The following equation (2) was used to determine the percentage of ItZ that penetrated the membrane and the overall amount of ItZ distributed throughout the receptor chamber (Hosny et al., 2021):

Eqn. (2) $$\mathrm{Percentage}\;\mathrm{permeated}=\frac{Lp}{LT}\;\times \;100$$

where LT was the initial concentration of ItZ in the donor chamber and Lp was the quantity of ItZ that was transferred into the receptor compartment.

##### 2.2.10.3. Assessment of the growth inhibition zone against Candida albicans

By using the previously indicated disk diffusion technique, the antifungal activity of the investigated formulations (F1 to F6), as well as the aqueous dispersion of ItZ, was assessed.

##### 2.2.10.4. Ulcer index evaluation

Male albino rats weighing 150 to 250 g were used for this investigation. The in-vivo study was performed according to the institutional guidelines of the Animal Ethics Committee of Cairo Agriculture for Experimental Animals, Cairo, Egypt, Approval No. (137-10-22). The ulcer index was assessed for seven animal groups, each consisting of three animals, using a previously described methodology (Alkhalidi et al., 2020). Group 2 received the ItZ aqueous suspension and was the positive control. Group 3 received formulation F1; Group 4 received the optimized nanoemulgel, or formula F2; Group 5 received formulation F4; Group 6 received formulation F5; and Group 7 received formulation F6. Group 1 received only normal saline and was the negative control.

After 3 days of therapy with each formulation, the ulcerative effect on the oral mucous membranes of every treated group was graded as 1 (normal-colored epithelial lining), 2 (red-colored epithelial lining), 3 (ulcers < 1 mm), 4 (ulcers > 1 mm but with no bleeding areas), and 5 (ulcers > 1 mm and with hemorrhagic streaks).

## 3. Results and discussion

### 3.1. Assessment of ItZ solubility

**Figure 1.**
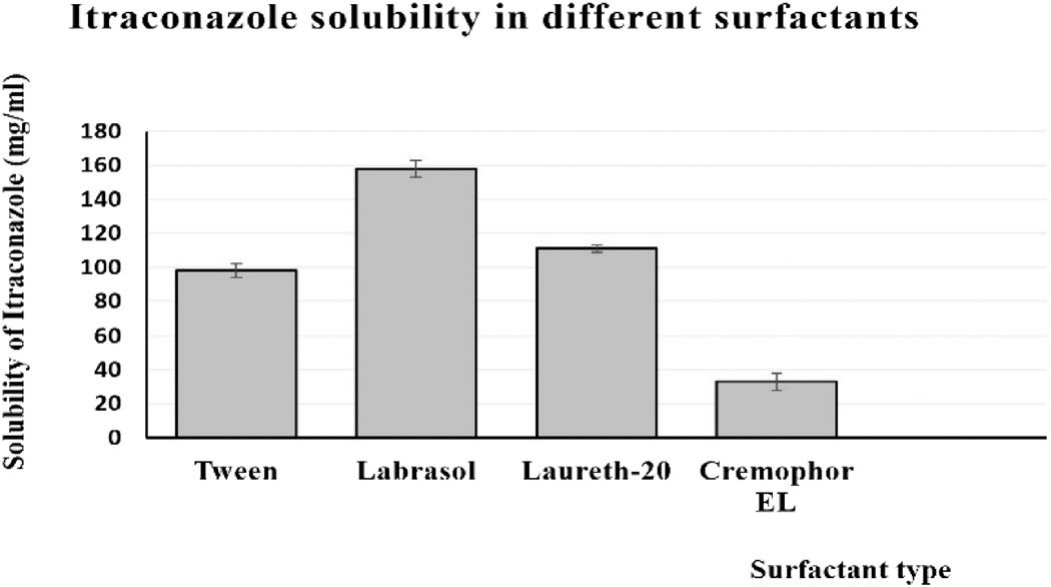
Solubilization properties of ItZ in various surfactants.

**Figure 2.**
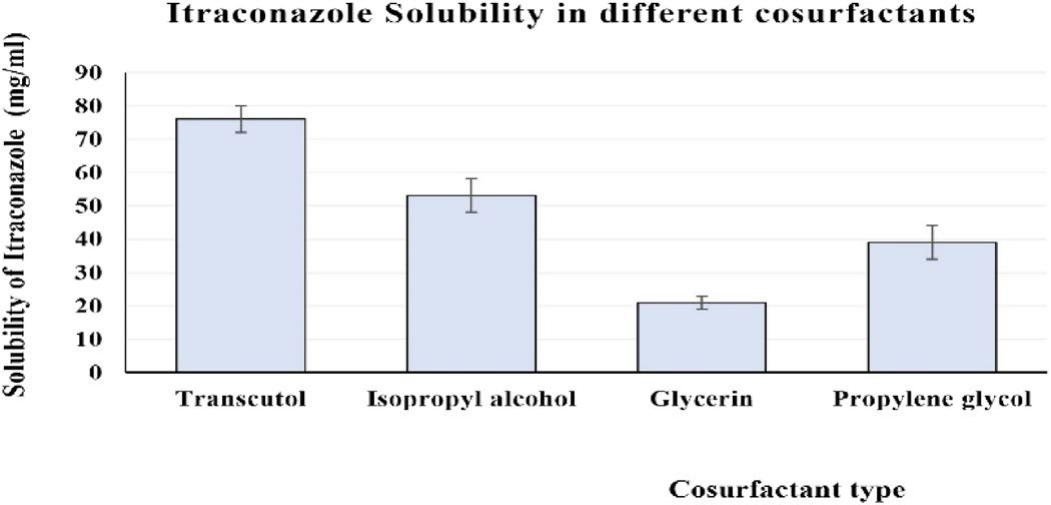
Solubilization properties of ItZ in various co-surfactants.

The ItZ had the best level of solubility when it was mixed with the surfactant labrasol and co-surfactant transcutol, according to the investigations into its solubility in various surfactants and co-surfactants. Figures 1 and 2 show the relationship between the types of surfactants and co-surfactants utilized and the percentage of ItZ solubilized. In order to determine the highest and lowest limits of each component of the SNEDDS, labrasol and transcutol were chosen for the pseudoternary phase diagram construction.

### 3.2. The ability of certain surfactants and co-surfactants to emulsify ItZ-ThO-EOs with a high degree of tenacity

In order to stabilize the system created by combining water and oil, a surfactant is frequently employed, with the goal of reducing the particle size. According to Tsai et al. (2014), the oil phase typically consists of natural triglycerides, hydrocarbons, and other nonpolar hydrophobic molecules, while the aqueous phase typically contains solutes and electrolytes that have been dissolved in water. To accurately pinpoint the range of concentrations of the thyme EO, labrasol, and transcutol that should produce regions of NE, a pseudoternary phase diagram was built for the current study.

**Figure 3.**
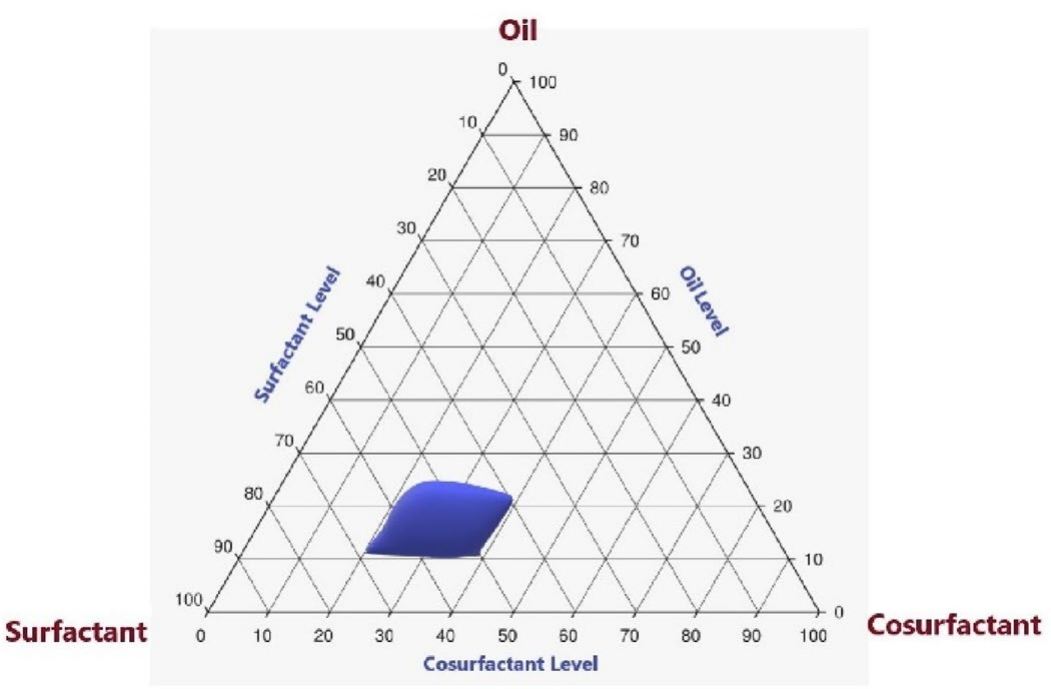
The pseudoternary phase plot of labrasol, transcutol, and thyme EO.

The precise concentration levels of thyme EO, labrasol, and transcutol for making NEs were found to be 10% to 25%, 40% to 70%, and 20% to 40%, respectively, as can be seen in the pseudoternary phase diagram in Figure 3. These concentration ranges were used to create the statistical design that was later used for the creation and optimization of several ItZ-ThO-SNEDDS formulations.

### 3.3. Visual examination of ItZ-ThO-SNEDDS

An examination of the ItZ-ThO-SNEDDS with the naked eye revealed translucid dispersions that were clear and did not exhibit any coalescence or breakage. This implied that the concentration ranges that were specified were exact in the establishment of stable NEs.

### 3.4. Assessment of ItZ-ThO-SNEDDS’ droplet size

The NEs had droplet sizes of 84 ± 2.1 to 240 ± 3.0 nm (Table 2) and PDI values between 0.09 and 0.32. This demonstrated the formulations’ uniformity, stability, and size distribution.

**Table 2.** Extreme vertices mixture design and the associated responses.

Run	A:ThO	B:Labrasol	C:Transcutol	Y_1_:Globule size (nm)	Y_2_: ItZ loading capacity (%)	Y_3_: Inhibition Zone (mm)	PDI
1	0.179	0.621	0.200	103 ± 1.6	63 ± 2.0	18 ± 0.7	0.11
2	0.169	0.536	0.294	143 ± 2.2	45 ± 1.0	9 ± 0.3	0.15
3	0.169	0.536	0.294	144 ± 1.9	46 ± 0.9	10 ± 0.5	0.12
4	0.192	0.471	0.336	151 ± 2.7	52 ± 1.3	12 ± 0.6	0.09
5	0.151	0.449	0.400	172 ± 2.5	41 ± 1.1	8 ± 0.4	0.10
6	0.169	0.536	0.294	142 ± 1.1	45 ± 0.5	9.5 ± 0.8	0.18
7	0.112	0.640	0.247	98 ± 0.9	40 ± 1.8	7.5 ± 0.3	0.28
8	0.250	0.485	0.264	188 ± 2.9	59 ± 0.4	16 ± 0.7	0.21
9	0.100	0.593	0.306	104 ± 3.1	39 ± 1.0	7 ± 0.5	0.25
10	0.100	0.700	0.200	84 ± 2.1	34 ± 0.3	6 ± 0.9	0.30
11	0.250	0.421	0.329	229 ± 3.3	57 ± 0.8	15 ± 0.4	0.32
12	0.179	0.621	0.200	102 ± 1.2	64 ± 1.1	20 ± 1.0	0.29
13	0.250	0.550	0.200	177 ± 1.8	61 ± 1.0	17 ± 0.3	0.22
14	0.100	0.500	0.400	161 ± 2.8	30 ± 1.5	5 ± 0.5	0.26
15	0.250	0.421	0.329	230 ± 3.9	56 ± 0.9	15 ± 0.1	0.17
16	0.118	0.538	0.344	138 ± 1.1	34 ± 0.3	6 ± 0.2	0.11
17	0.198	0.402	0.400	240 ± 3.0	54 ± 0.6	13 ± 0.6	0.19

The collected globule size data were subjected to a special quadratic model of polynomial analysis. The researched model was effective at determining the substantial impact of the thyme EO (A), labrasol (B), and transcutol (C) on the ItZ-ThO-SNEDDS’ droplet sizes, according to the chosen mathematical design. As shown in Table 3, the model had a modulated R^2^ value of 0.9991 and a predicted R^2^ value of 0.9960, which were closely correlated. An ANOVA data analysis produced the equation shown below:

**Table 3.** Regression analysis of the Y_1_, Y_2_, and Y_3_ responses.

Dependent variables	R^2^	Adjusted R^2^	Predicted R^2^	F-value	p-value	Adequate precision
Y_1_	0.9995	0.9991	0.9960	2203.64	0.0001	148.1595
Y_2_	0.9975	0.9950	0.9799	118.06	0.0001	58.9733
Y_3_	0.9871	0.9741	0.8931	26.68	0.0002	25.2220

*Droplet size = +527.85 A + 83.81 B + 262.16 C − 510.83 AB − 479.36 AC − 185.83 BC − 2689.11 A^2^BC + 3526.21 AB^2^C − 1622.86 ABC^2^* Eqn. (3)

**Figure 4.**
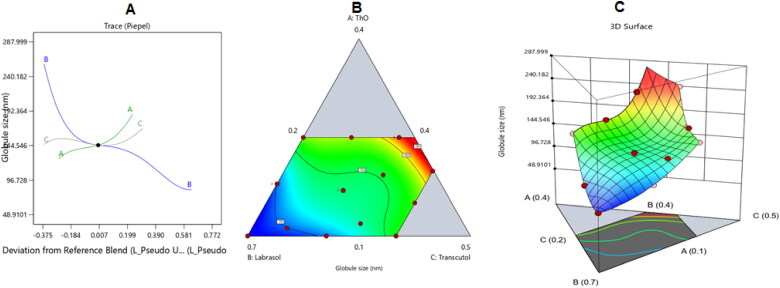
The impacts of various independent factors on the droplet size of various ItZ-ThO-SNEDDS are depicted in (A) a perturbation plot, (B) a contour plot, and (C) a 3D surface plot.

The perturbation, 3D surface, and contour plots in Figure 4 show how the parameters affected the size of the ItZ-ThO-SNEDDS droplets. The graphs demonstrate how the amount of different formulation variables in the formulations affected the globule size of the generated NEs.

According to the graph, labrasol (i.e. factor B) had the biggest impact on the droplets’ size. The increase in surfactant limits reduced the size of the globules, as shown in Figure 4(A), since this caused a lowering of the interfacial tension that existed between the aqueous and nonaqueous phases (Hosny et al., 2020). The fact that increasing the percentage of oil reduced the surfactant and co-surfactant levels and, therefore, limited their capacity to minimize the droplet size may help to explain the increase in droplet size in relation to the increased amount of ThO. Larger oil globules were produced as a result, and this was consistent with the outcomes mentioned in the literature (Ziani et al., 2011; Pavoni et al., 2020). Moreover, boosting the ThO level might have allowed for more ItZ, which is a lipophilic agent, to be incorporated into the oily droplets of the NE and, hence, for bigger droplets to be attained.

### 3.5. Assessment of ItZ incorporation efficiency within ItZ-ThO-SNEDDS

As demonstrated in Table 2, the loading of ItZ in the NEs ranged between 30 ± 1.5% and 64 ± 1.1%.

The resulting drug-loading data were subjected to a special quartic model of polynomial analysis. The ItZ-ThO-SNEDDS’ drug-loading capacity was evaluated using the model to assess the major impacts of ThO (A), labrasol (B), and transcutol (C). As shown in Table 3, the chosen model achieved an adjusted R^2^ value of 0.9950 and a forecasted R^2^ value of 0.9799. An ANOVA analysis of the data used the equation below:

*ItZ loading capacity = − 36.98 A + 34.34 B + 10.14 C + 248.22 AB + 267.58 AC + 55.50 BC + 792.65 A^2^BC − 1183.21 AB^2^C − 635.31 ABC^2^* Eqn. (4)

**Figure 5.**
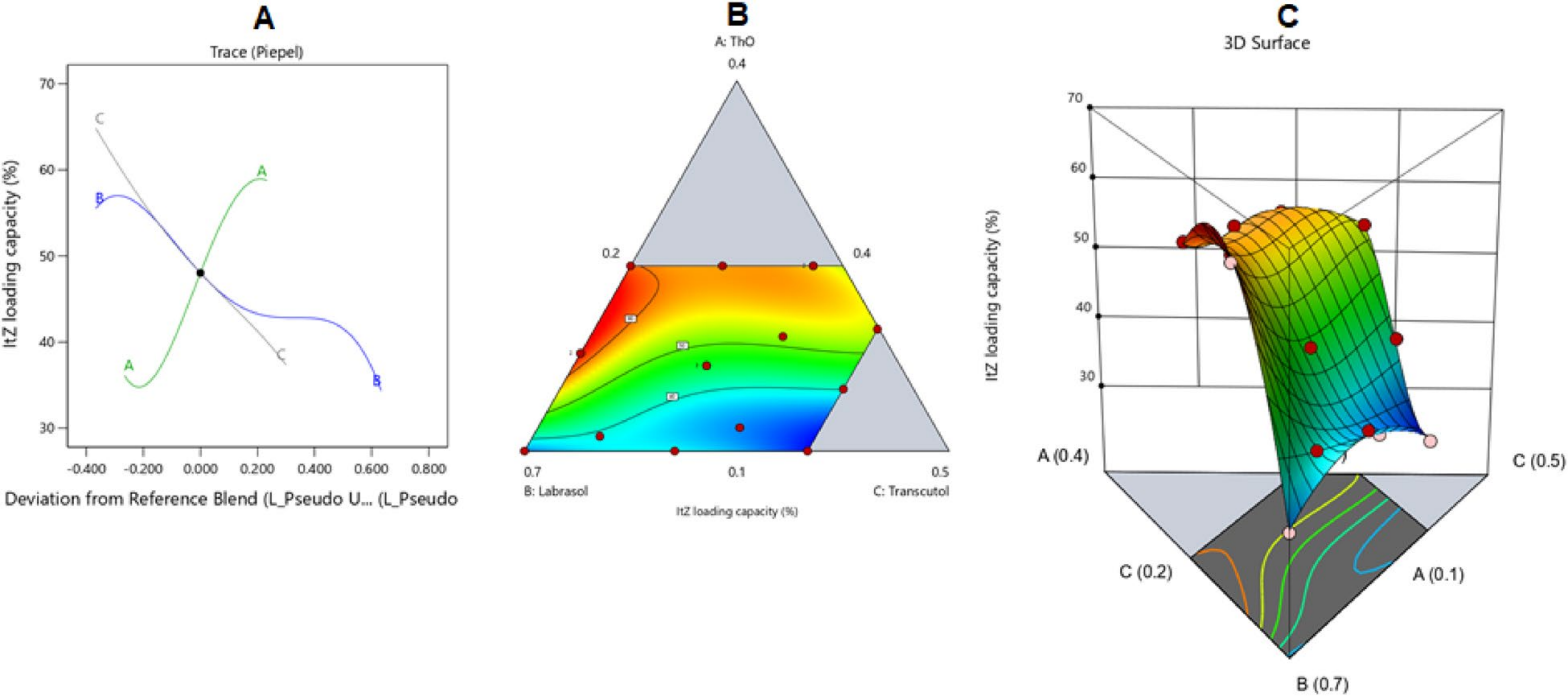
The impacts of various factors on the drug loading of various ItZ-ThO-SNEDDS are depicted in the (A) perturbation, (B) contour, and (C) 3D surface plots.

Figure 5 shows the main effect, contour, and 3D surface plots that illustrate how the investigated parameters affected the drug loading in the ItZ-ThO-SNEDDS.

As might be noticed from the preceding plots, the ThO level (i.e. factor A) significantly increased the incorporation of ItZ into the NE’s droplets. This finding might be due to the great capacity of the oil to absorb ItZ because of the lipophilic nature of ItZ. Further, improving the percentage of oil in the emulsion would lower the accompanying levels of labrasol and transcutol and, hence, lessen their ability to force the drug into the surrounding aqueous milieu (Hosny et al., 2021). Additionally, as can be seen from the graph, large amounts of labrasol or transcutol were associated with a lower loading capacity of ItZ in the NE. These outcomes could be ascribed to the comparable decrease in oil levels and, hence, the decreased space into which ItZ could be incorporated in the NE droplets. It could also be related to the interfacial tension–lowering properties of labrasol and transcutol.

### 3.6. Evaluation of fungal growth inhibition zones of ItZ-ThO-SNEDDS against Candida albicans

As is shown in Table 2, the inhibition zones against *C. albicans* created by the NE formulations ranged from 5 ± 0.5 to 20 ± 1.0 cm.

The zone inhibition data were fitted to a special quartic model of polynomial analysis. The ItZ-ThO-SNEDDS’ growth zone inhibition capabilities were examined using the selected statistical model to assess the major impacts of ThO (A), labrasol (B), and transcutol (C). As shown in Table 3, the chosen model had an adjusted R^2^ value of 0.9741 and a predicted R^2^ value of 0.8931. An ANOVA analysis of the data used the equation below:

*Inhibition Zone = − 23.62 A + 6.05 B − 2.54 C + 105.23 AB + 103.74 AC + 19.93 BC + 401.09 A^2^BC − 562.56 AB^2^C − 295.23 ABC^2^* Eqn. (5)

**Figure 6.**
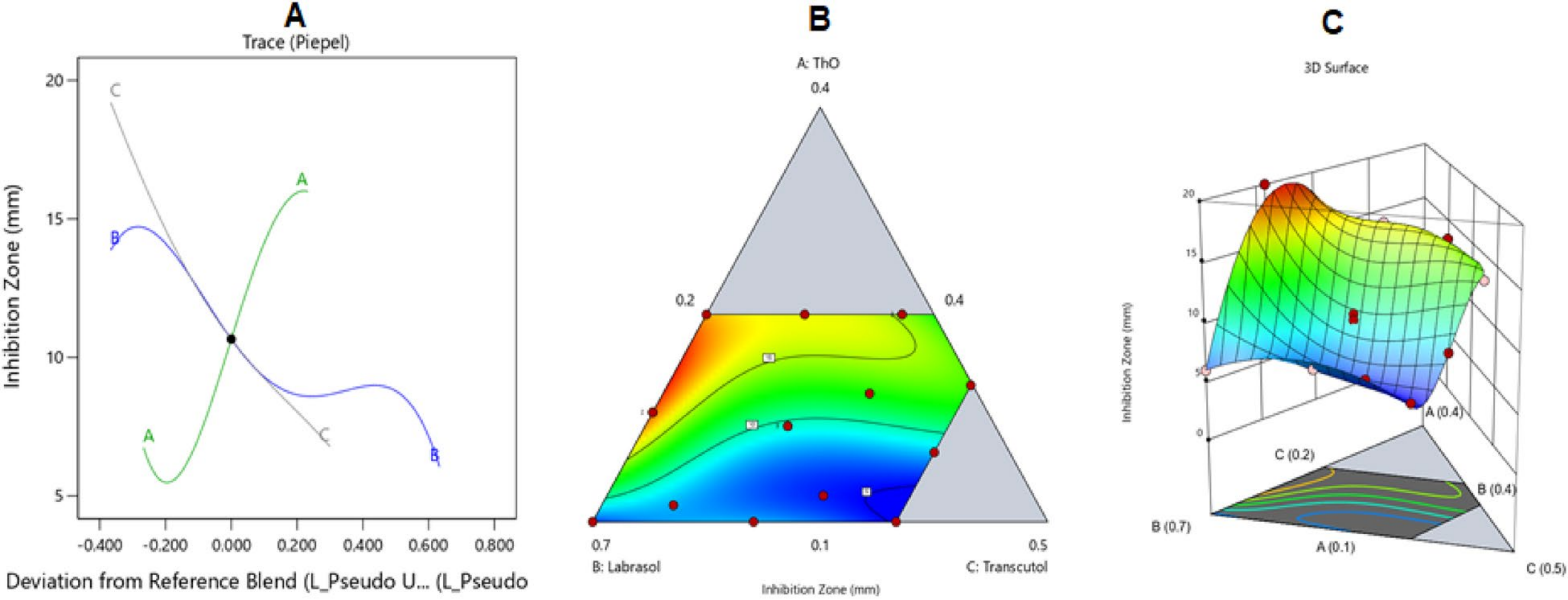
The impacts of various factors on the fungal growth inhibition zones of various ItZ-ThO-SNEDDS are depicted in the (A) perturbation, (B) contour, and (C) 3D surface plots.

Figure 6 shows the main effect, contour, and 3D surface graphs that illustrate how the investigated parameters impacted the fungal growth inhibition zones of ItZ-ThO-SNEDDS against *C. albicans*.

As might be seen from the above graphs, ThO has a great capacity for expanding the growth inhibition zones of *C. albicans*. Ergosterol is a special sterol that can only exist in the outer membrane of a fungus and is crucial to its healthy development and operation. As a result, substances affecting the level of ergosterol might have antifungal properties (Kowalczyk et al., 2020). Thymol, the major component of ThO, has potential antifungal action that is based on how it affects the metabolism of fatty acids, especially ergosterol, in fungal cells (De Lira Mota et al., 2012). Among other things, it produces oxidative stress and an increase in reactive oxygen species, and this lowers the levels of capsular polysaccharide and the extracellular polymer matrix (EPS) (Al-Shahrani et al., 2017). In cell membranes of *Candida* and *Cryptococcus* that had been exposed to thymol, ergosterol levels decreased, as previously stated in the literature. This resulted in disruptions of the membrane integrity and membrane-associated enzymes, significant damage, and, ultimately, cell death (Poonam et al., 2019).

The decrease in the size of the inhibition zones associated with the increase in labrasol (B) and transcutol (C) levels might be correlated with the comparable decrease in the level of ThO and, hence, its lowered ability to destroy the fungal cell membrane. The decrease in the area of the growth zone area observed when the surfactants were increased might be explained by the expected decrease in ItZ loaded into the NE and, hence, the decreased overall antifungal activities of the formulations.

### 3.7. Itz-ThO-SNEDDS optimization

**Figure 7.**
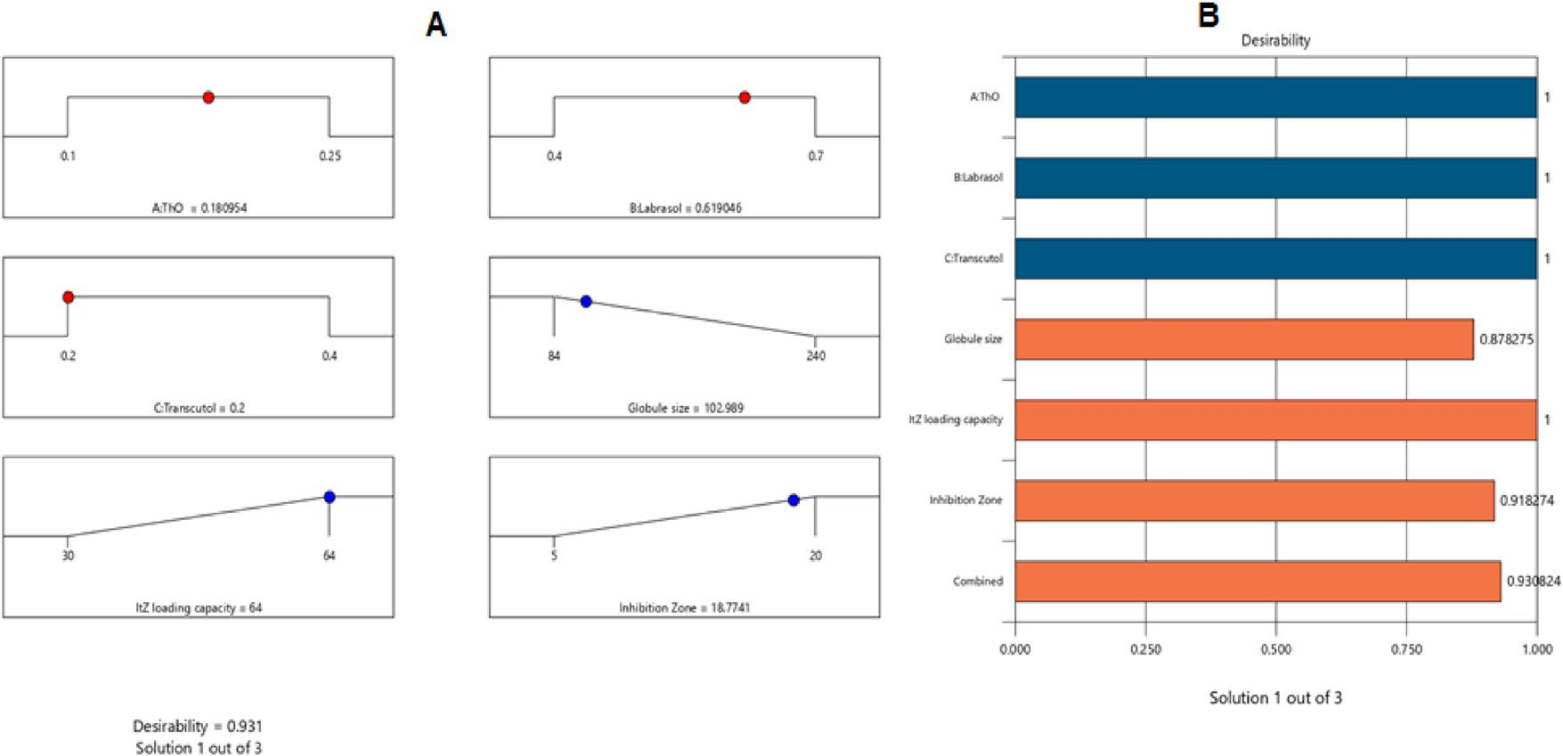
Bar chart and desirability ramp for process optimization. The levels of the parameters that were studied and the predicted estimations for the dependent variables of the optimal formulation.

The optimized NE formulation was created using the most appropriate qualities revealed by the aforementioned data. The best combination was composed of 0.18 g/ml of ThO, 0.62 g/ml of labrasol, and 0.2 g/ml of transcutol, according to Statgraphics software, which also suggested many other options that represented different combinations of the variables under study. The optimal formulation had a drug-loading capability of 67%, a droplet size of 105 nm, and an inhibition zone of 18.177 mm, and it acquired a desirability of 0.931. The desirability ramp is shown in Figure 7, which also shows the levels of the independent variables and the anticipated values of the measured responses for the best formulation. Table 4 shows that the parameters of the optimum formulation’s veritable and anticipated values were very nearly identical, with no appreciable discrepancies (*p* > .05), and this supported the accuracy, validity, and precision of the equations.

**Table 4.** Values obtained experimentally and realistically for the ideal NE formulation.

Solution	ThO	Labrasol	Transcutol	Droplet size (nm)	ItZ loading capacity(%)	Inhibition zone (mm)	desirability
Predicated value	0.1810	0.6190	0.2000	102.989	64	18.77	0.931
Experimental value	0.1810	0.6190	0.2000	105	67	18.00	0.931

### 3.8. In-vitro dissolution behavior of the ItZ-ThO-SNEDDS–loaded hydrogel formulations

**Figure 8.**
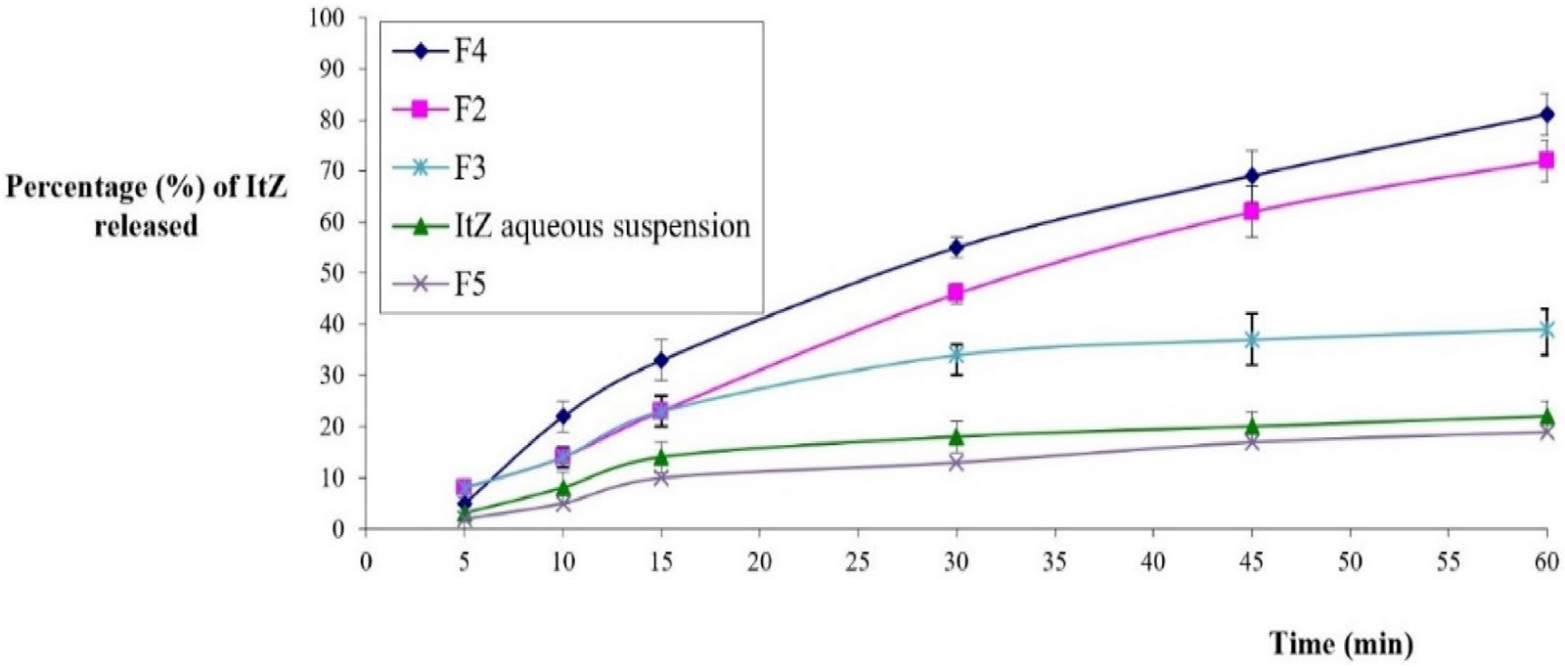
ItZ *in-vitro* release characteristics of the formulations.

The evaluated formulations’ drug release profiles are shown in Figure 8. The drug suspension had a very low cumulative percentage of the drug released (22 + 2%) after 60 minutes; this might be due to the low water solubility of ItZ. Formulation F4, which was a nanoemulgel without honey, had a drug release percentage of 85 ± 3%. Such a high percentage of release of the optimized formulation compared with the other examined formulations might be attributable to the NE content of amphiphilic molecules, which may improve drug release by enhancing ItZ dissolution (Hosny et al., 2021). Additionally, the small globule size of the NEs provided a broader surface area for drug release and aided in increasing the amount of drug released, enabling a higher amount of absorption and a quicker initiation of the drug’s effect (Hosny et al., 2021). In addition, the lack of honey in the formulation might have contributed to a decrease in the formulation’s viscosity, which could be expected to enhance the ItZ release. Meanwhile, formulation F2, which contained the optimal nanoemulgel and honey, had a drug release percentage of 72 ± 4%. The decrease in ItZ release from formulation F2 compared with formulation F4 could be due to the higher viscosity of the F2 with its honey content, which might have restrained the drug release (Salem et al., 2022). Formulation F3, which contained ItZ powder and ThO dispersed in a gel base, attained a percentage of release of 39 ± 4% of ItZ. Such a low release percentage may have resulted from poor ItZ solubility due to the lack of amphiphilic molecules in this formulation compared with the gel contained in the NE of same components (i.e. F4 and F2). Formulation F5, which contained ItZ powder in a gel base but no ThO, had a percentage of release of 19 ± 1% of ItZ. This low result might have been due to the presence of ItZ in a powder form but no surfactants that could have solubilized it, as well as to its viscous gel base, which further hindered its diffusion and release.

### 3.9. Ex-vivo transmucosal penetration test of the hydrogel formulations

As might be observed from Table 5, formulation F2, which was composed of the optimal formulation–loaded hydrogel that contained honey, achieved a Q_24_ value of 3125 μg/cm^2^ with a 4.16-fold increase in permeation compared with the ItZ aqueous suspension, which had a Q_24_ of 750 μg/cm^2^. Such a result demonstrates that the NEs were significantly involved in enhancing the ItZ penetration through the mucosa. The NEs’ vital role in increasing drug permeation can be ascribed to its content of surfactant, which might contribute to drug diffusion from the formulation and exert a considerable effect on fluidizing the mucosal cell membrane, leading to enhanced ItZ permeation (Salem et al., 2020). In addition, the small size of the NE globules provided a larger surface area for drug permeation. Simultaneously, formulation F4 had a Q_24_ of 2614 μg/cm^2^. Such a decrease in the Q_24_ compared with F4, even though both contained the ItZ NE, might be due to the presence of honey in F2. This could provide greater viscosity, which might have allowed for more intimate contact and a prolonged residence time of the formulation within the mucosa and, thus, a greater chance for ItZ to permeate the mucosal membrane (Laffleur, 2014). Moreover, F3, which contained ItZ powder and ThO dispersed in a gel base, had a Q_24_ of 1882 μg/cm^2^, while F5, which contained ItZ powder in a gel base but no ThO, had a Q_24_ of 800 μg/cm^2^. Such a large difference in the Q_24_ values of these two formulations may be attributable to the oil’s potential to increase permeability by altering and modifying cell membrane characteristics, such as by boosting ItZ solubility and penetration through the mucosa or by improving the membrane fluidity, as was previously described in the literature (Rizg et al., 2021; Jiang et al., 2017).

**Table 5.** *Ex-vivo* permeation parameters of ItZ through sheep buccal mucosa.

Parameters of permeation	F2	F4	F3	F5	ItZ Aqueous suspension
Cumulative amount permeated, Q_24_ (μg/cm^2^)^a^	3125	2614	1882	800	750
Steady state flux, Jss, (μg/cm^2^.min)^b^	52.4	44.9	31.4	16.2	18.8
Permeability coefficient, P, (cm/min)^c^	4.1 × 10^-3^	3.5 × 10^-3^	2.6 × 10^-3^	1.1 × 10^-3^	1.2 × 10^-3^
Diffusion coefficient, D, (cm^2^/min)^d^	2.03 × 10^-4^	1.72 × 10^-4^	1.21 × 10^-4^	0.58 × 10^-4^	0.51 × 10^-4^
Enhancement factor (F_en_)^e^	4.16	3.48	2.5	1.06	–

N.B. a: Q = the cumulative ItZ amount permeated thru membrane unit area; b: Jss = obtained from slop of curve plotted between Q and time; c: P = ItZ Jss/original concentration; d: D = Calculated from the slop of curve plotted between Q and square root of time; e: EF: Q of studied formulations/Q of ItZ suspension.

### 3.10. Ulcer index determination

The data in Table 6 show that F2 (i.e. the optimum ItZ-ThO-SNEDDS-loaded gel) had the lowest ulcer index. It was comparable to that of the negative control group, which received normal saline, and Group 3, which received plain gel. This finding revealed the substantially low ulcer-forming capacity of F2. Such noticeable activity might be ascribed to the nature of the gel base carrying F2, which had honey among its components. Honey has certain physical traits that encourage its application as a medicinal drug to fight certain infections (Hixon et al., 2019). These characteristics of honey are also connected to its ability to reduce inflammation and promote wound healing, antioxidant and the capacity to scavenge free radicals. It is an immune stimulator that can improve the immunological response (Almasaudi et al., 2016). It can be used to treat a variety of ulcers; it can also be utilized for wound healing and skin sanitation. In addition to its anti-inflammatory and bacteria-fighting capabilities, it accelerates the healing of wounds and reduces the ulcer index (Almasaudi et al., 2017).

**Table 6.** Ulcer index values of different animal groups treated with the tested formulations (means ± SD, *n* = 6).

Group treated	Tested Formulation	Ulcer Index
Group 1	Normal saline	1
Group 2	aqueous dispersion of ItZ	5
Group 3	formulation F1	1
Group 4	formulation F2	1
Group 5	formulation F4	3
Group 6	formulation F5	3
Group 7	formulation F6	2

N.B. F1: plain hydrogel; F2: optimum NE-loaded gel; F4: optimum NE-loaded gel but without honey; F5: gel loaded with ItZ powder and ThO; F6: gel containing only ThO.

These results were further confirmed when they were compared with the results for Group 2, which received the aqueous dispersion containing the drug and had an ulcer index of 5. Group 2 received ItZ twice daily for 14 days, and although ItZ has antifungal properties, because it is a synthetic drug it contributed to the injury of the buccal mucosal membrane. Groups 5 and 6, which received F4 and F5, respectively, both had ulcer indexes of 3. The change in the ulcer index for Groups 5 and 6 from that of Groups 3 and 4 might be due to the lack of honey in Group 5 (which received F4) or the lack of ThO in Group 6 (which received the gel containing ItZ powder only). Such outcomes emphasize the ulcer-reducing properties of both honey and ThO. The ulcer-healing activity of ThO was further proved by the results for Group 7, which received F6, which was composed of the gel loaded with NE but without ItZ; this group had an ulcer index of 2. This low ulcer index may have been due to the ThO content. ThO is well known for its anti-inflammatory and antioxidant properties, which contribute to its ability to heal lesions and ulcers; this is due to its main phenolic compounds, thymol and carvacrol (Nagoor Meeran et al., 2017; Kohlert et al., 2002).

## 4. Conclusion

ItZ was effectively developed as a functional NE in combination with ThO. A pseudoternary phase diagram was used to estimate the ideal concentrations of ThO, labrasol, and transcutol in combination to create the necessary drug delivery system. The created systems appeared to be reasonably stable, as evidenced by the NEs, which had globule sizes between 84 ± 2.1 and 240 ± 3.0 nm with acceptable homogeneous distribution. Additionally, the created formulations had a drug-loading efficiency of between 30 ± 1.5% and 64 ± 1.1% and a fungal growth inhibition zone of up to 20 mm. The extreme vertices mixture design was used to obtain the best formulation, which consisted of the variables ThO, labrasol, and transcutol at levels of 0.18 g/ml, 0.62 g/ml, and 0.2 g/ml respectively. The optimal formulation was loaded into a honey-based oral gel and showed enhanced release and transmucosal permeation. When tested in rats, the optimized ItZ-ThO-SNEDDS–loaded gel displayed the lowest ulcer index value when compared with other formulations. Overall, this study showed that ItZ-ThO–based NEs in combination with a honey-based gel could offer an effective defense against oral diseases brought on by microbial infections.

## References

[CIT0001] Alhakamy NA, Hosny KM, Aldryhim AY, et al. (2022). Development and optimization of ofloxacin as solid lipid nanoparticles for enhancement of its ocular activity. J Drug Deliv Sci Technol 72:1.

[CIT0002] Ali SA, Sindi AM, Mair YH, et al. (2021). Oral gel loaded by ethotransfersomes of antifungal drug for oral thrush: preparation, characterization, and assessment of antifungal activity. J Drug Deliv Sci Technol 66:102841.

[CIT0003] Ali SA, Sindi AM, Mair YH, Khallaf RA. (2021). Oral gel loaded by ethotransfersomes of antifungal drug for oral thrush: preparation, characterization, and assessment of antifungal activity. J Drug Delivery Sci Technol 66:102841.

[CIT0004] Alkhalidi HM, Hosny KM, Rizg WY. (2020). Oral gel loaded by fluconazole–sesame oil nanotransfersomes: development, optimization, and assessment of antifungal activity. Pharmaceutics 13:27. 33375740 10.3390/pharmaceutics13010027 PMC7823766

[CIT0005] Alkhalidi HM, Naguib GH, Kurakula M, et al. (2018). In vitro and preclinical assessment of factorial design based nanoethosomal transdermal film formulation of mefenamic acid to overcome barriers to its use in relieving pain and inflammation. J Drug Del Sci Technol 48:450–12.

[CIT0006] Almasaudi SB, Abbas AT, Al-Hindi RR, et al. (2017). Manuka honey exerts antioxidant and anti-inflammatory activities that promote healing of acetic acid-induced gastric ulcer in rats. Evid Based Complement Alternat Med 2017:5413917. 28250794 10.1155/2017/5413917 PMC5307292

[CIT0007] Almasaudi SB, El-Shitany NA, Abbas AT, et al. (2016). Antioxidant, anti-inflammatory, and antiulcer potential of manuka honey against gastric ulcer in rats. Oxid Med Cell Longev 2016:3643824. 26770649 10.1155/2016/3643824 PMC4685122

[CIT0008] Al-Shahrani MH, Mahfoud M, Anvarbatcha R, et al. (2017). Evaluation of antifungal activity and cytotoxicity of *Thymus vulgaris* essential oil. PC 7:34–40.

[CIT0009] Bendif H, Peron G, Miara MD, et al. (2020). Total phytochemical analysis of *Thymus munbyanus* subsp. coloratus from Algeria by HS-SPME-GC-MS, NMR and HPLC-MSn studies. J Pharmaceut Biomed 186:113330. 10.1016/j.jpba.2020.113330 32371324

[CIT0010] Buset SL, Walter C, Friedmann A, et al. (2016). Are periodontal diseases really silent? A systematic review of their effect on quality of life. J Clin Periodontol 43:333–44. 26810308 10.1111/jcpe.12517

[CIT0011] Chang YL, Yu SJ, Heitman J, et al. (2017). New facets of antifungal therapy. Virulence 8:222–36. 27820668 10.1080/21505594.2016.1257457 PMC5354158

[CIT0012] Chong CR, Sullivan DJ. Jr. (2007). New uses for old drugs. Nature 448:645–6. 17687303 10.1038/448645a

[CIT0013] Dangi YS, Soni MS, Namdeo KP. (2010). Oral candidiasis: a review. Int J Pharm Pharm Sci 2:36–41.

[CIT0014] De Lira Mota KS, de Oliveira Pereira F, de Oliveira WA, et al. (2012). Antifungal activity of *Thymus vulgaris* L. essential oil and its constituent phytochemicals against *Rhizopus oryzae*: interaction with ergosterol. Molecules 17:14418–33. 23519243 10.3390/molecules171214418 PMC6268362

[CIT0015] DiMasi JA, Hansen RW, Grabowski HG. (2003). The price of innovation: new estimates of drug development costs. J Health Econ 22:151–85. 12606142 10.1016/S0167-6296(02)00126-1

[CIT0016] Eva L, Christin M, Ahmed M, et al. (2018). Authorised medicinal product Aspecton® Oral Drops containing thyme extract KMTv24497 shows antiviral activity against viruses which cause respiratory infections. J Herb Med 13:26–33.

[CIT0017] FDI World Dental Federation. (2015). The challenge of oral diseases. 2nd ed. Brighton, UK: Myraid Editions.

[CIT0018] Ghasemi G, Alirezalu A, Ghosta Y, et al. (2020). Composition, antifungal, phytotoxic, and insecticidal activities of *Thymus kotschyanus* essential oil. Molecules 25:1152. 32143475 10.3390/molecules25051152 PMC7179150

[CIT0019] Girois SB, Chapuis F, Decullier E, et al. (2006). Adverse effects of antifungal therapies in invasive fungal infections: review and meta-analysis. Eur J Clin Microbiol Infect Dis 25:138–49. 16622909 10.1007/s10096-005-0080-0

[CIT0020] Gleiter R, Horn H, Isengard H. (2006). Influence of type and state of crystallisation on the water activity of honey. Food Chem 96:441–5.

[CIT0021] Gregg Stetsko Manager (1986). Statistical experimental design and its application to pharmaceutical development problems. Drug Develop Indust Pharm 12:1109–23.

[CIT0022] Guo S, Dipietro LA. (2010). Factors affecting wound healing. J Dent Res 89:219–29. 20139336 10.1177/0022034509359125 PMC2903966

[CIT0023] Hixon KR, Klein RC, Eberlin CT, et al. (2019). A critical review and perspective of honey in tissue engineering and clinical wound healing. Adv Wound Care 8:403–15. 10.1089/wound.2018.0848 PMC6855288 31737423

[CIT0024] Hosny K, Asfour H, Rizg W, et al. (2021). Formulation, optimization, and evaluation of oregano oil nanoemulsions for the treatment of infections due to oral microbiota. Int J Nanomed 16:5465–78. 10.2147/IJN.S325625 PMC8370598 34413644

[CIT0025] Hosny KM, Alhakamy NA, Sindi AM, et al. (2020). Coconut oil nanoemulsion loaded with a statin hypolipidemic drug for management of burns: formulation and in vivo evaluation. Pharmaceutics 12:1061. 33171816 10.3390/pharmaceutics12111061 PMC7695003

[CIT0026] Hosny KM, Khallaf RA, Asfour HZ, et al. (2021). Development and optimization of cinnamon oil nanoemulgel for enhancement of solubility and evaluation of antibacterial, antifungal and analgesic effects against oral microbiota. Pharmaceutics 13:1008. 34371700 10.3390/pharmaceutics13071008 PMC8309164

[CIT0027] Hosny KM, Rizg WY, Khallaf RA. (2020). Preparation and optimization of in situ gel loaded with rosuvastatin-ellagic acid nanotransfersomes to enhance the anti-proliferative activity. Pharmaceutics 12:263. 32183144 10.3390/pharmaceutics12030263 PMC7151021

[CIT0028] Hosny KM, Sindi AM, Alkhalidi HM, et al. (2021). Development of omega-3 loxoprofen-loaded nanoemulsion to limit the side effect associated with NSAIDs in treatment of tooth pain. Drug Deliv 28:741–51. 33840320 10.1080/10717544.2021.1909179 PMC8057080

[CIT0029] Hussein RM, Kandeil MA, Mohammed NA, Khallaf RA. (2022). Evaluation of the hepatoprotective effect of curcumin-loaded solid lipid nanoparticles against paracetamol overdose toxicity: Role of inducible nitric oxide synthase. J Liposome Res 8:1–11. 10.1080/08982104.2022.2032737 35132919

[CIT0030] Jiang Q, Wu Y, Zhang H, et al. (2017). Development of essential oils as skin permeation enhancers: penetration enhancement effect and mechanism of action. Pharm Biol 55:1592–600. 28399694 10.1080/13880209.2017.1312464 PMC7011944

[CIT0031] Katz J, Wallet S, Cha S. (2010). Periodontal disease and the oral-systemic connection: “is it all the RAGE?”. Quintessence Int 41:229–37. 20213024

[CIT0032] Kohlert C, Schindler G, März RW, et al. (2002). Systemic availability and pharmacokinetics of thymol in humans. J Clin Pharmacol 42:731–7. 12092740 10.1177/009127002401102678

[CIT0033] Kosakowska O, Bączek K, Przybył JL, et al. (2020). Morphological and chemical traits as quality determinants of common thyme (*Thymus vulgaris* L.), on the example of ‘StandardWinter’ cultivar. Agronomy 10:909.

[CIT0034] Kowalczyk A, Przychodna M, Sopata S, et al. (2020). Thymol and thyme essential oil-new insights into selected therapeutic applications. Molecules 25:4125. 32917001 10.3390/molecules25184125 PMC7571078

[CIT0035] Laffleur F, Keckeis V. (2020). Advances in drug delivery systems: work in progress still needed? Int J Pharm X 2:100050. 32577616 10.1016/j.ijpx.2020.100050 PMC7305387

[CIT0036] Laffleur F. (2014). Mucoadhesive polymers for buccal drug delivery. Drug Dev Ind Pharm 40:591–8. 24576266 10.3109/03639045.2014.892959

[CIT0037] Langemo DK, Hanson D, Anderson J, et al. (2009). Use of honey for wound healing. Adv Skin Wound Care 22:113–8. 19247011 10.1097/01.ASW.0000305460.87058.42

[CIT0038] Molan P, Rhodes T. (2015). Honey: a biologic wound dressing. Wounds 27:141–51. 26061489

[CIT0039] Molan PC. (2001). Potential of honey in the treatment of wounds and burns. Am J Clin Dermatol 2:13–9. 11702616 10.2165/00128071-200102010-00003

[CIT0040] Molan PC. (2002). Re-introducing honey in the management of wounds and ulcers – theory and practice. Ostomy Wound Manage 48:28–40. 12426450

[CIT0041] Nagoor Meeran MF, Javed H, Taee HA, et al. (2017). Pharmacological properties and molecular mechanisms of thymol: Prospects for its therapeutic potential and pharmaceutical development. Front Pharmacol 8:380. 28694777 10.3389/fphar.2017.00380 PMC5483461

[CIT0042] Nilima T, Silpi B, Rakesh NB, et al. (2013). Antimicrobial efficacy of five essential oils against oral pathogens: an in vitro study. Eur J Dent 7:71–7. 10.4103/1305-7456.119078 PMC4054083 24966732

[CIT0043] Okabayashi K, Imaji M, Osumi T, et al. (2009). Antifungal activity of itraconazole and voriconazole against clinical isolates obtained from animals with mycoses. Nihon Ishinkin Gakkai Zasshi 50:91–4. 19430183 10.3314/jjmm.50.091

[CIT0044] Parihar S. (2011). Oral candidiasis – a review. Webmedcentral Dent 2:1–18.

[CIT0045] Pavoni L, Perinelli DR, Bonacucina G, et al. (2020). An overview of micro- and nanoemulsions as vehicles for essential oils: formulation, preparation and stability. Nanomaterials (Basel 10:135. 31940900 10.3390/nano10010135 PMC7023169

[CIT0046] Poonam K, Neha A, Apurva C, et al. (2019). Delineating the biofilm inhibition mechanisms of phenolic and aldehydic terpenes against *Cryptococcus neoformans*. ACS Omega 4:17634–48. 31681870 10.1021/acsomega.9b01482 PMC6822124

[CIT0047] Prasanna KR. (2012). Oral candidiasis – a review. Scholarly J Med 2:6–30.

[CIT0048] Rajan B, Ahmed J, Shenoy N, et al. (2014). Assessment of quality of life in patients with chronic oral mucosal diseases: a questionnaire-based study. Perm J 18:e123-7. 24626087 10.7812/TPP/13-095 PMC3951046

[CIT0049] Rizg WY, Hosny KM, Elgebaly SS, et al. (2021). Preparation and optimization of garlic oil/apple cider vinegar nanoemulsion loaded with minoxidil to treat alopecia. Pharmaceutics 13:2150. 34959435 10.3390/pharmaceutics13122150 PMC8706394

[CIT0050] Salehi B, Mishra AP, Shukla I, et al. (2018). Thymol, thyme, and ­other plant sources: health and potential uses. Phytother Res 32:1688–706. 29785774 10.1002/ptr.6109

[CIT0051] Salem HF, El-Menshawe SF, Khallaf RA, et al. (2020). A novel transdermal nanoethosomal gel of lercanidipine HCl for treatment of hypertension: optimization using Box-Benkhen design, in vitro and in vivo characterization. Drug Deliv Transl Res 10:227–40. 31625026 10.1007/s13346-019-00676-5

[CIT0052] Salem HF, Nafady MM, Ewees MGE, et al. (2022). Rosuvastatin calcium-based novel nanocubic vesicles capped with silver nanoparticles-loaded hydrogel for wound healing management: optimization employing Box-Behnken design: in vitro and in vivo assessment. J Liposome Res 32:45–61. 33353435 10.1080/08982104.2020.1867166

[CIT0053] Sell SA, Wolfe PS, Spence AJ, et al. (2012). A preliminary study on the potential of manuka honey and platelet-rich plasma in wound healing. Int J Biomater 2012:1–14. 10.1155/2012/313781 PMC3523149 23304152

[CIT0054] Sharifi-Rad M, Varoni EM, Iriti M, et al. (2018). Carvacrol and human health: A comprehensive review. Phytother Res 32:1675–87. 29744941 10.1002/ptr.6103

[CIT0055] Silva AE, Demarco FF, Feldens CA. (2015). Oral healthrelated quality of life and associated factors in Southern Brazilian elderly. Gerodontology 32:35–45. 23848341 10.1111/ger.12050

[CIT0056] Sundoro A, Nadia K, Nur A, et al. (2012). Comparison of physical–chemical characteristic and antibacterial effect between Manuka honey and local honey. J Plastik Rekonstruksi 1:3.

[CIT0057] Talat M, Zaman M, Khan R, et al. (2021). Emulgel: an effective drug delivery system. Drug Dev Ind Pharm 47:1193–9. 34647512 10.1080/03639045.2021.1993889

[CIT0058] Terezhalmy GT, Huber MA. (2011). Oropharyngeal candidiasis: etiology, epidemiology, clinical manifestations, diagnosis, and treatment. Crest Oral‑B at Dentalcare.com Contin Educ Course 1–16.

[CIT0059] Thimmaraju MK, Pamulaparthy V, Raghunandan N. (2012). Development and validation of RP-HPLC method for the determination of itraconazole in bulk and capsule dosage form. J Anal Chem 2:10.

[CIT0900] Tsai M-J, Fu Y-S, Lin Y-H, Huang Y-B, Wu P-C. (2014). The effect of Nanoemulsion as a carrier of Hydrophilic compound for transdermal delivery. PLoS ONE 9:e102850. 10.1371/journal.pone.0102850 PMC4113283 25068531

[CIT0060] Walther C, Döring K, Schmidtke M. (2020). Comparative in vitro analysis of inhibition of rhinovirus and influenza virus replication by mucoactive secretolytic agents and plant extracts. BMC Complement Med Ther 20:380. 33357221 10.1186/s12906-020-03173-2 PMC7757078

[CIT0061] Wong RSH, Dodou K. (2017). Effect of drug loading method and drug physicochemical properties on the material and drug release properties of poly (ethylene oxide) hydrogels for transdermal delivery. Polymers (Basel) 9:286. 30970963 10.3390/polym9070286 PMC6432290

[CIT0062] World Health Organization. Oral health fact sheet. (2012). Available at: http://www.who.int/ mediacentre/factsheets/fs318/en/

[CIT0063] Ziani K, Chang Y, McLandsborough L, et al. (2011). Influence of surfactant charge on antimicrobial efficacy of surfactant-stabilized thyme oil nanoemulsions. J Agric Food Chem 59:6247–55. 21520914 10.1021/jf200450m

